# Cannabidiol extending beyond neuroprotection toward neuronal repair: A potential regenerative modulator

**DOI:** 10.4103/NRR.NRR-D-25-01176

**Published:** 2025-12-30

**Authors:** María Salud García-Gutiérrez, Jorge Manzanares

**Affiliations:** Instituto de Neurociencias, Universidad Miguel Hernández-CSIC, San Juan de Alicante, Alicante, Spain; Red de Investigación en Atención Primaria de Adicciones, Instituto de Salud Carlos III, MICINN and FEDER, Madrid, Spain; Instituto de Investigación Sanitaria y Biomédica de Alicante (ISABIAL), Alicante, Spain

Cannabidiol (CBD), the second most significant phytocannabinoid in the plant Cannabis sativa, which lacks potential as a drug of abuse (Viudez-Martinez et al., 2019), has gained widespread attention due to its anti-inflammatory, antioxidant, and antidepressant properties (Garcia-Gutierrez et al., 2020). Additionally, CBD exhibits neuroprotective properties, preserving neuronal viability and function by preventing or limiting cellular damage. Our team has demonstrated that CBD produces rapid antidepressant-like effects in a murine model of chronic mild stress, restoring hippocampal expression of brain-derived neurotrophic factor (BDNF), serotonin 1A (5-HT_1A_), and peroxisome proliferator-activated receptor delta (PPARδ), and surpassing the effects of the conventional selective serotonin reuptake inhibitor sertraline. Similarly, CBD promotes hippocampal neurogenesis and enhances synaptic plasticity (Garcia-Gutierrez et al., 2023). Besides, CBD restores hippocampal neurodegeneration in a mouse model of fetal alcohol spectrum disorder (Gasparyan et al., 2023).

Although cumulative evidence suggests the neuroprotective properties of CBD, emerging data reveal its ability to enhance regenerative processes by influencing progenitor proliferation, axonal sprouting, remyelination, synaptic reorganization, and glial phenotype modulation. We propose positioning CBD as a pro-regenerative modulator capable of enhancing repair in the central nervous system, beyond its protective role. This perspective explores potential mechanisms through which CBD promotes regeneration, including the modulation of neurotrophic factors and key signaling pathways, such as cannabinoid receptors, peroxisome proliferator-activated receptors (PPARs), and transient receptor potential vanilloid (TRPV) channels, as well as glial reprogramming.

By shifting the paradigm from symptomatic neuroprotection to tissue repair and functional recovery, CBD could open new therapeutic avenues in regenerative neurology.

**The multifaceted and distinctive mechanism of cannabidiol:** Since the characterization of this cannabinoid compound in 1963 (Mechoulam and Shvo, 1963), cumulative evidence emphasizes its complex and unique mechanism that underscores its impressive therapeutic potential. CBD uniquely influences over 65 biological targets, including critical receptors such as 5-HT_1A_, transient potential vanilloid 1 (TRPV1), and peroxisome proliferator-activated gamma (PPARγ), demonstrating its broad spectrum of activity.

Despite ongoing debates, substantial evidence confirms that CBD functions as a negative allosteric modulator of the cannabinoid 1 receptor (CB_1_r), not directly, but by inhibiting fatty acid amide hydrolase, thereby elevating anandamide levels. Additionally, CBD acts as an inverse agonist of the cannabinoid 2 receptor (CB_2_r), which contributes to its therapeutic effects. Furthermore, CBD’s action on vital targets involved in neuroinflammation, such as the adenosine receptor 2A, adenosine transporter 1, and the P2X7 purinergic receptor, highlights its promising role in addressing complex psychiatric and neurological conditions.

Several studies have demonstrated that CBD exhibits a wide range of pharmacological properties, including antioxidant, anti-inflammatory, anxiolytic, antidepressant, antiepileptic, and neuroprotective effects, which promote its research in psychiatry and neurology (Garcia-Gutierrez et al., 2020). While CBD’s neuroprotective capacity was initially noted, recent research indicates its therapeutic potential for stimulating regenerative processes by acting on progenitor proliferation, synapse remodeling, axonal sprouting, remyelination and glial phenotype modulation, supporting structural and functional recovery.

**Evidence supporting the impact of cannabidiol on neurogenesis and synapse remodeling:** Preclinical evidence, as well as human studies to a lesser extent, reveal the powerful neuroprotective effects of CBD. Interestingly, CBD promotes neurogenesis and synapse remodeling, fundamental processes for brain repair.

Our research group showed that CBD effectively normalizes and partially repairs neuronal damage in a murine model of fetal alcohol spectrum disorder, underscoring its restorative capabilities (Gasparyan et al., 2023). Furthermore, in an additional study, we demonstrated that chronic administration of CBD significantly enhances hippocampal neuronal survival and elevates BDNF levels in mice subjected to chronic stress, highlighting its potential to restore stress-related neuronal damage (Garcia-Gutierrez et al., 2023). Collectively, these compelling studies strongly suggest that CBD is a potent agent supporting endogenous neural repair, especially under conditions where neurogenesis is compromised by stress, drug toxicity, aging, or neurodegenerative processes.

CBD mediates these effects, acting on cannabinoid receptors, PPARγ, and TRPV1, not only modulating inflammatory gene expression but also significantly enhancing calcium homeostasis and progenitor cell proliferation. Besides, activation of the 5-HT_1A_ receptor further amplifies these benefits by increasing progenitor cell proliferation and upregulating BDNF, thereby strongly supporting neural growth (Zanelati et al., 2010; Campos et al., 2012). Extensive scientific research confirms that CBD activates multiple key pathways, including extracellular signal-regulated kinase 1/2 (ERK1/2)-cAMP response element-binding protein (CREB), glycogen synthase kinase 3 beta, postsynaptic density protein 95, and phosphoinositide 3-kinase (PI3K)/mammalian target of rapamycin (mTOR)/p70S6K, which are integral to cell survival and synaptic plasticity. These findings underscore powerful potential of CBD as a new drug for brain regeneration and cognitive resilience, making this cannabinoid a promising candidate for therapeutic development.

Beyond this broad pharmacological profile, specific signaling pathways appear to be more directly linked to regenerative effects of CBD. Among these, the ERK/CREB and PI3K/mTOR/p70S6K cascades are central to survival and synaptic plasticity, while BDNF-TrkB signaling plays a crucial role in neurogenesis and dendritic remodeling. Although anti-inflammatory actions of CBD can facilitate these processes, several findings suggest that CBD also exerts regenerative effects independent of glial modulation, directly enhancing progenitor proliferation and synaptic reorganization. Additionally, antioxidant properties of CBD, modulation of TRPV channels, and influence on endocannabinoid signaling may contribute to a pro-regenerative microenvironment. In this regard, CBD may be positioned alongside other regenerative modulators, such as neurotrophins, erythropoietin, and stem-cell-derived factors; however, its pleiotropic activity on multiple receptor systems and signaling pathways may represent a distinctive advantage.

**Evidence supporting the impact of cannabidiol on gliosis and microglial activation:** Several studies suggest that CBD stimulates axonal sprouting and facilitates synaptic reconnection. These actions occur by effectively controlling reactive gliosis and persistent microglial activation that creates a hostile environment for neural regeneration. Treatment with CBD restores neuroinflammatory markers, such as interleukin-1β and tumor necrosis factor-α, induces a beneficial switch in microglia from the pro-inflammatory M1 state to the repair-oriented M2 state, and reduces astrocyte glial fibrillary acidic protein expression, thereby creating a permissive environment for axonal sprouting, synaptic reconnection, and network repair. These effects have been consistently confirmed across various *in vivo* models of neurodegenerative diseases, including Alzheimer’s disease, Parkinson’s disease, and multiple sclerosis (Echeverry et al., 2025). Interestingly, synthetic CBD derivatives with improved bioavailability have been shown to modulate the immune response in multiple sclerosis, thereby favoring neuroprotection and axonal regeneration (Navarrete et al., 2018, 2020). These studies have indicated that CBD derivatives exhibit anti-inflammatory activity, prevent demyelination, and enhance remyelination.

These convincing findings further suggest that CBD or derivatives could be a potent therapeutic agent for improving neural recovery and fighting neurodegeneration.

**Evidence supporting the impact of cannabidiol on synaptic plasticity and dendritic remodeling:** CBD enhances synaptic plasticity and dendritic remodeling, processes that are essential for learning, memory, and long-term functional recovery following injury or degeneration in the central nervous system. In models of epilepsy and Alzheimer’s disease, CBD restores long-term potentiation at least by activation of PPARγ (Hughes and Herron, 2019). CBD also promotes the expression of synaptic scaffolding proteins such as the postsynaptic density protein 95, synapsin I, and growth-associated protein 43, encouraging synaptogenesis, dendritic spine branching, and network connectivity (Santos et al., 2015; Xie et al., 2024). These findings correlate with improvements in behavior and emotional responses. Collectively, these actions highlight dual role of CBD in neuroprotection and regeneration.

In animal models of Alzheimer’s disease and various models of neural injury and dysfunction, CBD improves cognitive outcomes in memory tasks and prevents synaptic loss. Interestingly, our research expands this knowledge by demonstrating that CBD restores cognitive impairment associated with depression, increasing BDNF expression and neuronal nuclei immunoreactivity in the hippocampus (Garcia-Gutierrez et al., 2023).

These findings suggest that CBD contributes to the restoration of the neural network by promoting synaptic resilience and dendritic complexity. Evidence supports that CBD stimulates the rebuilding of neuronal connections, reinforcing its potential as a regenerative modulator in the treatment of neurodegenerative and neuropsychiatric disorders.

**Positioning cannabidiol within regenerative neurology:** Despite the promising data from preclinical studies, there are some gaps in our understanding of the regenerative potential of CBD. The majority of data comes from rodent models, and robust evidence in humans remains limited. Therefore, a cautious interpretation of preclinical findings is necessary before extrapolating to human applications.

Moreover, the heterogeneity of research designs, dosing regimens evaluated, and outcome measures makes the interpretation and comparison of results across the studies difficult. Results can vary between experiments, reflecting differences in models, treatment duration, and experimental conditions. Moreover, it is necessary to elucidate further the mechanisms underlying the effects of CBD. Addressing these gaps is crucial for advancing CBD from preclinical studies to clinical applications.

CBD is currently approved for treating spasticity and pain in multiple sclerosis, as well as for seizures related to Lennox-Gastaut and Dravet syndromes. In recent years, the commercial availability of CBD oils and supplements has increased, leading to widespread use by patients seeking relief from various conditions, such as chronic pain, anxiety, migraines, and inflammation. Additionally, many countries have legalized CBD for medical use. However, this has raised concerns about safety and effectiveness.

It is important to note that over-the-counter CBD products lack regulatory oversight, resulting in inconsistencies in purity, dosage, and concentration.

Furthermore, the long-term safety of chronic CBD use is not well understood, nor is its interaction with standard medications such as antiepileptics, anticoagulants, or antidepressants. In this regard, our study showed that combining CBD with the antidepressant sertraline worsened treatment outcomes when controlling depressive-like symptoms (Garcia-Gutierrez et al., 2023). On the other hand, the combination of CBD with sertraline resulted in a synergistic effect, enhancing fear extinction and reducing anxiety-like behaviors in a mouse model of post-traumatic stress disorder (Gasparyan et al., 2021).

Additionally, differences in formulation, poor oral bioavailability, and individual metabolic variability complicate the establishment of standardized therapy. Therefore, despite its potential benefits, there is an urgent need for controlled clinical trials, pharmacovigilance, and the development of standardized pharmaceutical-grade products.

**Figure 1:**
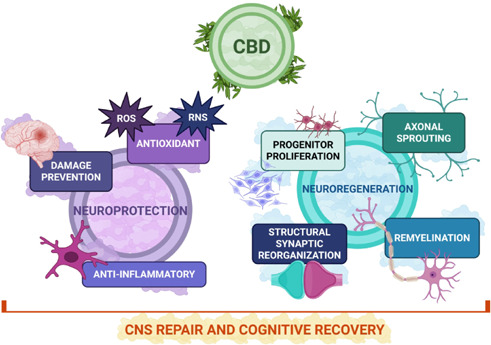
Schematic representation of the proposed dual role of CBD in neuroprotection and neuroregeneration. CBD influences multiple molecular targets and signaling pathways that support both neuroprotective and neuroregenerative properties. Neuroprotective actions are suggested to include the limitation of neuronal damage through anti-inflammatory and antioxidant mechanisms. Potential regenerative actions involve processes such as progenitor proliferation, axonal sprouting, remyelination, and synaptic reorganization, which contribute to structural repair. Together, these complementary processes are hypothesized to favor CNS repair and cognitive recovery. Created with BioRender.com. CBD: Cannabidiol; CNS: central nervous system; RNS: reactive nitrogen species; ROS: reactive oxygen species.

**Concluding remarks and future directions:** In summary, promising results support the hypothesis that CBD may act as a regenerative modulator and a promoter of cognitive recovery in injuries and neurodegenerative disorders in the central nervous system (**Figure 1**). Future studies should incorporate cognitive endpoints, such as learning, memory, and executive function, into regenerative models of neurological disorders, e.g., stroke and traumatic brain injury.

Further research employing translational models with regeneration-specific endpoints, such as axonal sprouting, remyelination, and synaptic reorganization, would substantially improve our understanding of role of CBD in neural repair.

Standardized, long-term clinical trials with CBD could reveal whether improvements in neurotrophic signaling, neurogenesis, and synaptic plasticity driven by CBD lead to measurable functional and cognitive recovery in humans. Finally, future studies may also explore CBD analogues or derivatives with improved bioavailability, which could enhance translational applicability.

*This work was supported by Instituto de Salud Carlos III, Spanish Ministry of Science and Innovation, grant number PI18/00576 to MSGG and JMR and Red de Investigación en Atención Primaria de Adicciones, Instituto de Salud Carlos III, Spanish Ministry of Science and Innovation, grant number RD21/0009/0008 and RD24/0003/0002, and Instituto de Investigación Sanitaria y Biomédica de Alicante (ISABIAL) to JM. The Instituto de Neurociencias is a “Centre of Excellence Severo Ochoa” (CEX2021-001165-S).*
